# Support of Home-Based Structured Walking Training and Prediction of the 6-Minute Walk Test Distance in Patients With Peripheral Arterial Disease Based on Telehealth Data: Prospective Cohort Study

**DOI:** 10.2196/65721

**Published:** 2025-04-10

**Authors:** Fabian Wiesmüller, Andreas Prenner, Andreas Ziegl, Gihan El-Moazen, Robert Modre-Osprian, Martin Baumgartner, Marianne Brodmann, Gerald Seinost, Günther Silbernagel, Günter Schreier, Dieter Hayn

**Affiliations:** 1 Center for Health & Bioresources, AIT Austrian Institute of Technology GmbH, Graz, Austria; 2 Ludwig Boltzmann Institute for Digital Health and Prevention, Ludwig Boltzmann Gesellschaft, Salzburg, Austria; 3 Institute of Neural Engineering, Graz University of Technology, Graz, Austria; 4 Division of Angiology, Department of Internal Medicine, Medical University of Graz, Graz, Austria; 5 telbiomed Medizintechnik und IT Service GmbH, Graz, Austria

**Keywords:** mHealth, telehealth, peripheral arterial disease, home-based structured walking training, trend estimation, predictive modeling, continuous data, walking, walking training, prediction, prediction model, cardiovascular disease, stroke, heart failure, physical fitness, telehealth system

## Abstract

**Background:**

Telehealth has been effective in managing cardiovascular diseases like stroke and heart failure and has shown promising results in managing patients with peripheral arterial disease. However, more work is needed to fully understand the effect of telehealth-based predictive modeling on the physical fitness of patients with peripheral arterial disease.

**Objective:**

For this work, data from the Keep Pace study were analyzed in depth to gain insights on temporal developments of patients’ conditions and to develop models to predict the patients’ total walking distance at the study end. This could help to determine patients who are likely to benefit from the telehealth program and to continuously provide estimations to the patients as a motivating factor.

**Methods:**

This work analyzes continuous patient-reported telehealth data, in combination with in-clinic data from 19 Fontaine stage II patients with peripheral arterial disease who underwent a 12-week telehealth-based walking program. This analysis granted insights into the increase of the total walking distance of the 6-minute walk tests (6MWT) as a measure for physical fitness, the steady decrease in the patients’ pain, and the positive correlation between well-being and the total walking distance measured by the 6MWT.

**Results:**

This work analyzed trends of and correlations between continuous patient-generated data. Findings of this study include a significant decrease of the patients’ pain sensation over time (*P*=.006), a low but highly significant correlation between pain sensation and steps taken on the same day (*r*=−0.11; *P*<.001) and the walking distance of the independently performed 6MWTs (*r*=−0.39; *P*<.001). Despite the reported pain, adherence to the 6MWT measurement protocol was high (85.53%). Additionally, patients significantly improved their timed-up-and-go test times during the study (*P*=.002). Predicting the total walking distance at the study end measured by the 6MWT worked well at study baseline (root mean squared error of 30 meters; 7.04% of the mean total walking distance at the study end of 425 meters) and continuously improved by adding further telehealth data. Future work should validate these findings in a larger cohort and in a prospective setting based on a clinical outcome.

**Conclusions:**

We conclude that the prototypical trend estimation has great potential for an integration in the telehealth system to be used in future work to provide tailored patient-specific advice based on these predictions. Continuous data from the telehealth system grant a deeper insight and a better understanding of the patients’ status concerning well-being and level of pain as well as their current physical fitness level and the progress toward reaching set goals.

## Introduction

### Background

Peripheral arterial disease is a significant global health concern with an estimated 202 million people affected worldwide [1]. Prevalence in Europe is reported at 5.3%, increasing to 15%‐20% among individuals over the age of 70 years [2]. Peripheral arterial disease is characterized by insufficient oxygen supply due to arterial vessel alterations in the extremities. This leads to pain when walking, forcing patients to stop walking and thus affecting mobility and quality of life. Pain may occur even at rest. Furthermore, the healing of wounds is slowed down, and peripheral arterial disease may ultimately lead to necrosis. Peripheral arterial disease is classified into distinct grades according to the Rutherford system, which includes 6 categories, and the Fontaine classifications, which include 4 stages. Evidence-based guidelines for the management of peripheral arterial disease are provided by organizations such as the European Society of Cardiology, European Society of Vascular Medicine, and American Heart Association [3-5].

Treatment for Fontaine stage I is typically limited to risk factor management (eg, nicotine abstention and blood pressure management), which remains a relevant factor throughout all following stages. In Fontaine stage II, medication regimen and structured walking training (SWT) become additional treatment options. While medication is also recommended throughout stages III and IV, SWT is only prescribed in stages II and III. Stages III and IV have surgical interventions as an additional treatment option [3,4].

SWT can more than double the pain-free walking distance of patients with peripheral arterial disease within a 12-week program [6,7], and achieve long-term outcomes comparable to surgical intervention (revascularization) while being less expensive and non-invasive [4,8]. To further foster SWT in patients with peripheral arterial disease, the Trans-Atlantic Inter-Society Consensus-II group has defined a training protocol for patients. This recommendation suggests performing walking exercises at an intensity that induces pain for 3‐5 minutes in total within a 30‐60-minute session. Such a session should be conducted 3 times per week for 3 months [9]. To evaluate the progress of SWT and to assess the physical fitness of patients with peripheral arterial disease, standardized tests have been developed.

The state-of-the-art method for measuring the physical fitness of patients with peripheral arterial disease is the 6-minute walk test (6MWT), during which a patient walks as far as possible within 6 minutes on an incline-free surface [10]. Usually, the 6MWT is performed under the supervision of a health care professional (HCP) who measures the total walking distance after the completion of the test [11]. Another test to measure physical fitness is the timed-up-and-go (TUG) test. The TUG test starts with the participant sitting on a chair. The participant is then instructed to stand up, walk for 3 meters, turn around, walk back, and sit back down. The time the participant needs to complete this sequence of tasks is measured [12]. Even though TUG tests are not primarily used for patients with peripheral arterial disease, they provide information about the patients’ general mobility and frailness. Additionally, a previous publication identified a significant correlation between the 6MWTs and TUG tests in patients with peripheral arterial disease [13].

Telehealth is defined as the delivery of health care or rehabilitation services to patients remotely using digital technologies [14], which, over the last years, has proven to be effective in the field of cardiovascular diseases, particularly heart failure and stroke [15,16]. Telehealth and telerehabilitation solutions have recently also shown promising results in the field of peripheral arterial disease [17,18]. Telehealth can support early detection of (post-operative) complications, with the potential to improve functional capacity, claudication onset time, and quality of life for patients with peripheral arterial disease [19].

Despite these studies, the benefits and the potential of telehealth in the setting of peripheral arterial disease are not yet fully explored. Aspects like the influence of continuously measured parameters and digital tools on the physical fitness of patients with peripheral arterial disease must be further investigated.

### Keep Pace Study Protocol

During the Keep Pace study, 19 patients with peripheral arterial disease underwent a 12-week program with digital assistance to evaluate the impact of telehealth solutions on patients with peripheral arterial disease with a focus on the decision of undergoing surgery after the program [20]. The participants for the Keep Pace study were recruited at the outpatient clinics of the Department of Angiology of the University Hospital Graz, Austria. Patients who were shortlisted for getting an angioplasty were contacted and asked to participate in the Keep Pace study. Patients who gave informed consent and fit the inclusion criteria were included in this study. The inclusion criteria were as follows: age between 18 and 80 years, peripheral arterial disease in Fontaine stage IIa or IIb, ownership of an Android mobile device, and capability to use telehealth services. Patients lacking compliance to perform SWT were excluded. At the onset of the study, an initial in-clinic visit was performed, during which the patient’s medical history and subjective limitations in daily activities, including pain-free and total walking distance, were assessed. All patients were diagnosed with peripheral arterial disease based on imaging diagnostics before study entrance. Because the location and grade of the occlusion were not part of the analysis, all diagnostic imaging options, such as duplex ultrasound, computer tomography, and magnetic resonance angiography, were accepted, which did not provide information about the location and grade of the occlusion for all patients. In addition to the imaging peripheral arterial disease criteria, claudication had to be present for patients to be eligible for this study. In the following 8 weeks participants were telemedically supervised by a nurse. In the subsequent remaining 4 weeks, patients performed their SWT therapy independently without regular telehealth consultations. Thereafter, an in-clinic study end visit was performed [20] .

During the Keep Pace study, the collected telehealth data have been used to continuously monitor the patients’ status. They might also be used for predictive modeling applications, eg, to detect already at early stages, which patients will benefit from the training and which patients might require additional attention. It is expected that such a prediction could help to achieve a more patient-specific therapeutic regime, tailored exercise support, and expectation management. However, currently, there is no evidence to support this hypothesis. This work takes an in-depth look at the telehealth data recorded during the Keep Pace study [20] by focusing on analyzing trends in patient-reported timeseries data.

### Objective

The aim of this investigation was to test the feasibility of predicting the total 6-minute walk test distance at the study end in patients with peripheral arterial disease, which can be used to determine patients who will likely benefit more from the telehealth program than others and additionally provide patient-specific feedback and training regime adaptions throughout the patients’ time in the telehealth system.

## Methods

### Dataset

For this work, data of 19 patients with peripheral arterial disease in the Fontaine stage II were analyzed, which were collected over a 12-week study period in the Keep Pace study [20]. While the study protocol only specified that participants had to be patients with peripheral arterial disease in Fontaine stage II, the actual patient cohort was unanimously in Fontaine stage IIb. The characteristics of the patients are shown in Table 1.

Data from 2 sources were collected during Keep Pace and further processed in this analysis: (1) data assessed in-clinic by HCPs at the beginning and at the end of the study (Textbox 1) and (2) patient-reported data collected with a telehealth system during the 12-week study period (Textbox 2).

**Table 1.** Patient characteristics by gender and in total (SD) [20].

Characteristics		Female (n=7)	Male (n=13)	Total (n=20)
Age, years, mean (SD)		64.14 (8.68)	62.46 (5.39)	63.05 (6.55)
Weight, kg, mMean (SD)		70.14 (9.5)	81.67 (11.95)	77.64 (12.27)
Height, m, mean (SD)		1.65 (0.03)	1.76 (0.05)	1.72 (0.07)
BMI, kg/m²		25.79 (3.44)	26.33 (4.51)	26.15 (4.08)
Subjective pain-free walking distance, m, mean (SD)		73.57 (50.22)	81.54 (37.16)	78.75 (41.04)
Fontaine stage IIa, n		0 (0%)	0 (0%)	0 (0%)
Fontaine stage IIb, n		7 (100%)	13 (100%)	20 (100%)
Diabetics, n (%)		1 (14%)	2 (15%)	3 (15%)
Hypercholesterolemia, n (%)		7 (100%)	13 (100%)	20 (100%)
Smokers, n (%)		2 (28%)	10 (77%)	12 (60%)
Coronary artery disease, n (%)		3 (43%)	4 (31%)	7 (35%)

Textbox 1. **In-clinic data.**

Patient characteristics: sex, height, age, weight, disease-related data like the date of diagnosis, cardiovascular risk factors, current medication, diabetes and smoking assessment and year of diagnosis

6MWTs: manual measurement by an HCP and automated measurement with a dedicated 6MWT app [21]

TUG test data: automated measurement with a TUG test device as described in [21,22], including the total TUG time and sub-task times (stand-up, walk-forward, turnaround, walk-back, sit-down, total walk time, and mobility time)

Textbox 2. **Patient-reported telehealth data.**

6MWTs: automated measurement with a 6MWT app [21] — recommended once per week

Steps per day: automatically measured by the mobile phone

Subjective well-being: recommended once per day

Pain: recommended once per day

The telehealth data described in (Textbox 2) were gathered by the patients using a dedicated mobile application. Patients did not receive an incentive for completing the 6MWTs and the SWT apart from the inherent incentive of improving their physical fitness and quality of life by adhering to the measurement and training regime. Subjective well-being was assessed on a 3-item scale (good, medium, and bad) and pain was reported on a scale (0=no pain to 10=maximum pain). The steps per day were recorded with the smartphones’ native step counter, and patients could perform unsupervised 6MWTs using an integrated previously developed GPS-based 6MWT-app [21].

### Predictive Modeling

We investigated different models for predicting the total walking distance at the study end right at the onset of the program (baseline) and at week *n* of the program, only based on data recorded until week *n*. A leave-one-out setting was applied, which excluded 1 patient from the training process and used this patient as a validator for the developed algorithm, resulting in 19 different data splits per tested model. This ensures that each patient is used as the validator once per model. The quality of the prediction as compared to the measured data at the study end was calculated using the root mean squared error (RMSE). The following predictive models were applied.

#### Distance at Baseline

This approach was mainly used as a reference for other models. The 6MWT distance at the study end was estimated as equal to the 6MWT distance at baseline (ie, this model assumed no improvement during the study).

#### Average Improvement Based on All Patients

This model assumed that all patients had similar improvements. Therefore, for estimating the 6MWT distance at the study end of patient *i* in week *n* (*d_av,n,i_*), the mean change of all other patients from week *n* to the study end was calculated and added to the respective patient’s 6MWT distance *d_n,i_* in week *n* (equation 1).


(1) $$d_{av,n,i}=\;d_{n,i}+\;\frac{1}{18}\sum_{\begin{array}{c} j=1\\ j\neq i \end{array}}^{19}\left(d_{end,\;j}-d_{n,j}\right)$$

#### Logarithmic Interpolation Based on an Individual Patient

Logarithmic interpolation was used to fit a logarithmic function as defined by the parameters *a_n,i_* and *b_n,i_* into all measured 6MWT distances *d_k,j_* performed at week *x_k,j_* until week *n* by the respective patient *i*. The estimated distance at the study end as derived in week *n* for patient *i* (*d_log_ind,n,i_*) was calculated according to equation 2.


(2) $$d_{log\_ind,n,i}=(a_{n,i}*\log_{e}x+b_{n,i}){|}_{x=12}$$

$$with\left[a_{n,i},b_{n,i}\right]=logfit\left(x_{k,j},d_{k,j}\right)\;{|}_{\forall k\leq n,\;j=i}$$

#### Logarithmic Interpolation Based on All Patients

Assuming that all patients improved similarly, logarithmic interpolation was also applied to data from all patients when defining parameters *a_n_* and *b_n_* in week *n*. Therefore, all measurements *d_k,j_* were first corrected for the mean of each patient ($\overset{-}{d_{k,j}}$) (see equation 3).


(3) $$d_{log\_all,n,i}=(a_{n,i}*\log_{e}x+b_{n,i}{)|}_{x=12}$$

$$with\left[a_{n},b_{n}\right]=logfit\left(x_{k,j},\overset{-}{d_{k,j}}\right)\;{|}_{\forall k\leq n,\forall j}$$

##### Linear Regression

All models described earlier are based on 6MWT distances only. With the linear regression approach, also other data assessed during the program were considered. Feature candidates applied for predictions in week *n* were derived from all data recorded during the in-clinic visit at baseline (see In-clinic data in the Methods section) and from telehealth data recorded until week *n*. Telehealth data contained the feature candidates, as shown in Textbox 3:

All possible combinations of up to 8 features were evaluated. Therefore, each feature was first tested on its own, and subsequently features were added iteratively to test all possible combinations. However, due to the small sample size, a maximum number of 4 features was the goal.

Textbox 3. **Feature candidates from telehealth data.**

Mean 6MWT distance up to week *n*: this parameter represents all total walking distance measurements from the 6MWT app from the baseline up to the week of the prediction.

Mean 6MWT distance in week *n*: this parameter considers only measurements of 6MWTs performed in the week of the prediction.

Logarithmic prediction in week *n*: this feature is derived from the trend-based analysis, where a predicted outcome is available for each week.

### Statistical Analyses

All statistical analyses were implemented in MATLAB (The MathWorks) and all predictive models were either implemented in MATLAB or Python. Wherever reasonable, pre- and post-study comparisons were performed on data assessed at the in-clinic visits at baseline and the study end, using paired tests. Continuously measured parameters were tested for correlation with one another. Unless stated otherwise, correlations were tested using the Pearson’s correlation coefficient with a previous Anderson-Darling test to test for a normal distribution of the data. Trend analyses were performed for all the continuous measurements mentioned earlier for which the data were aggregated on a weekly basis by calculating mean values per parameter. Predictive models were evaluated by calculating the root mean square error between the estimated 6MWT total walking distance at the study end and the actually measured distance. For this work, a *P* value <.05 was considered to be significant.

### Ethical Considerations

Ethical approval for the study was obtained from the Ethics Committee of the Medical University of Graz (34‐127 ex 21/22 1566‐2021, ClinicalTrials.gov Identifier: NCT05619835). All analyses performed during this work are covered by this ethics approval. All participants received oral and written information prior to the study entry and provided written informed consent to participate in this study. This informed consent also covers the analysis of secondary study outcomes performed in this paper. All data were recorded in a pseudonymized way and anonymity was ensured for all participants during the presentation of the findings. Patients did not receive any financial incentives for participating in the study.

## Results

### Data Characteristics and Trend Analysis

#### The 6-Minute Walk Test

The study protocol recommended 12 6MWTs. 57.89% (11 out of 19) patients performed 12 or more tests and were 100% adherent to the measurement protocol. The 8 remaining patients performed, on average, 7.88 (SD 2.36) 6MWTs and hence achieved a mean adherence of 65.62% (SD 19.64%) of the 12 recommended tests. On average, patients performed 13.37 (SD 8.90) 6MWTs and overall adherence to the measurement routine was 85.53% (SD 21.31%).

**Figure 1.**
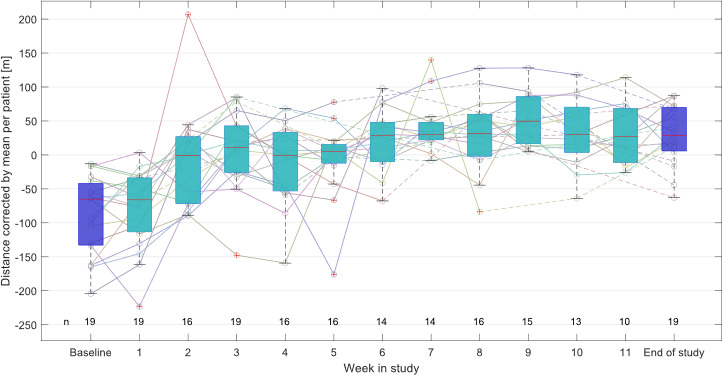
Course over time of weekly aggregated 6-minute walk test (6MWT) total walking distances for all patients, illustrated as lines per patient and boxplots. Data are normalized for the patient-individual mean. Dashed lines illustrated interpolations over weeks with missing values. In-clinic measurements of the first and last visit are shown in dark blue; tests measured with the 6MWT app are shown in azure. The number n of patients who performed at least 1 6MWT in the respective week is shown on the bottom.

Figure 1 illustrates the 6MWT total walking distance aggregated on a weekly basis for all patients. The total walking distance increased over time, following a logarithmic trend.

As can be seen in Table 2, a significant correlation was found between the 6MWT total walking distance at baseline versus the distance at the study end. However, no significant correlation between either the number of tests and the distance at baseline, or the number of tests and the distance at the study end, or the number of tests and the change in walking distance during the program, or between the distance at baseline and the change in walking distance during the program has been identified.

**Table 2.** Correlation between the final walking distance and the number of tests and the change in the walking distance between baseline and the study end (R: correlation coefficient; PC: Pearson correlation coefficient; S: Spearman correlation coefficient).

	Distance at the study end	Number of tests	Change in walking distance
Distance at baseline	*r*=0.78, *P*<.001, S	*R*=–0.14, *P*=.56, S	*r*=0.02, *P*=.93, S
Distance at the study end	N/A	*r*=−0.17, *P*=.48, S	*r*=0.71, *P*<.001, S
Number of tests	*r*=−0.13, *P*=.60, PC	N/A	*r*=0.02, *P*=.93, S

#### Timed-Up-and-Go Test

Table 3 summarizes all TUG subtask measures at baseline and the study end. Table 3 shows that the sit-down time significantly decreased from 2.21 to 1.89 seconds (*P*=.038), and the total walk time significantly decreased from 6.96 to 6.57 seconds (*P*=.003). Additionally, the overall TUG time significantly decreased from 10.6 seconds at baseline to 9.89 seconds at the study end (*P*=.002). Also, all other parameters decreased but did not reach statistical significance.

**Figure 2.**
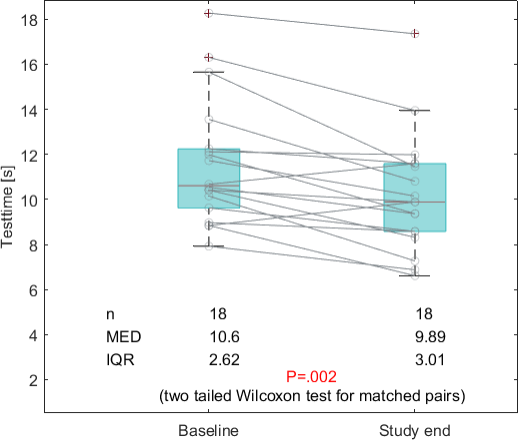
Improvement of the timed-up-and-go test time at baseline versus the study end (n: number of observations; MED: median; IQR: interquartile range).

Figure 2 shows the decrease of the total TUG time.

**Table 3.** TUG subtask times (in seconds; median (IQR)) at baseline and the study end with the *P* values calculated with the 2-tailed Wilcoxon test for matched pairs.

	Baseline, median (IQR)	Study end, median (IQR)	*P* value
Stand-up	2.67 (2.34-4.44)	2.54 (2.10-3.00)	.09
Walk-forward	1.95 (1.70-2.21)	1.76 (1.69-2.10)	.06
Turnaround	1.82 (1.43-2.34)	1.56 (1.15-2.08)	.11
Walk-back	2.09 (1.95-2.35)	1.95 (1.82-2.21)	.06
Sit-down	2.21 (2.10-2.87)	1.89 (1.56-2.60)	.04
Total walk time	6.96 (5.98-9.78)	6.57 (5.33-7.42)	.003
Mobility time	4.10 (3.77-4.43)	3.64 (3.51-4.04)	.08
Total TUG time	10.6 (9.63-12.25)	9.89 (8.58-11.59)	.002

#### Steps Per Day

The mean number of steps per day as measured by the smartphone’s native app was 4176.58 (SD 2742.73). No significant change in steps per day was found when aggregating over all patients. However, 3 patients showed a significant reduction of steps, and 4 patients significantly improved. No relation between the change of recorded steps and the decision of undergoing surgery after the program was observed.

#### Pain

**Figure 3.**
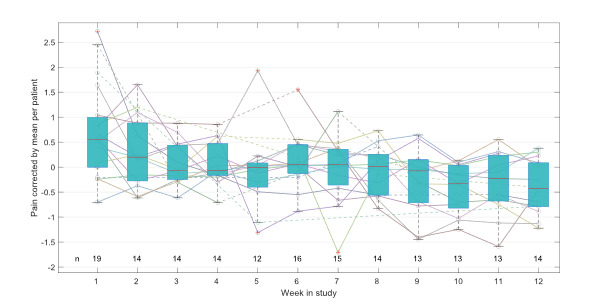
Course over time of weekly aggregated pain measurements for all patients, illustrated as lines per patient and boxplots. The level of pain is normalized for the patient-individual mean. Dashed lines illustrated interpolations over weeks with missing values. The number of patients who reported at least 1 pain level in the respective week is shown on the bottom.

Overall, patient-reported pain was significantly reduced from a mean of 4.67 (SD 1.90) at baseline to 3.60 (SD 2.39) at the study end (*P*<.05). Additionally, pain showed a very low but highly significant correlation with steps per day (*r*=−0.11; *P*<.001) and the distance of the 6MWTs (*r*=−0.39; *P*<.001) on days when patients submitted both measurements. Figure 3 illustrates the course over time of weekly pain values corrected for the mean per patient.

#### Subjective Well-Being

Out of 1597 subjective well-being data points, 41 (2.63%) were reported as bad*,* 686 (43.0%) as medium, and 870 (54.5%) as good. No trends or patterns in the development of subjective well-being and no significant change from baseline to the study end were identified. On 5 days with bad, 160 with medium, and 106 with good well-being, 6MWTs were performed by the respective patient. On days with a submitted subjective well-being and a 6MWT, the number of bad well-being was 1.88%, which is in the same magnitude as the overall bad well-being submissions. Subjective well-being showed a significant correlation with the 6MWT total walking distance (*r*=0.26, *P*<.001, Spearman). On days with subjective well-being and step count data available (40 bad, 657 medium, and 835 good), a weak but highly significant correlation between subjective well-being and steps per day was found (*r*=0.19; *P*<.001).

### Trend Prediction and Predictive Modeling

**Figure 4.**
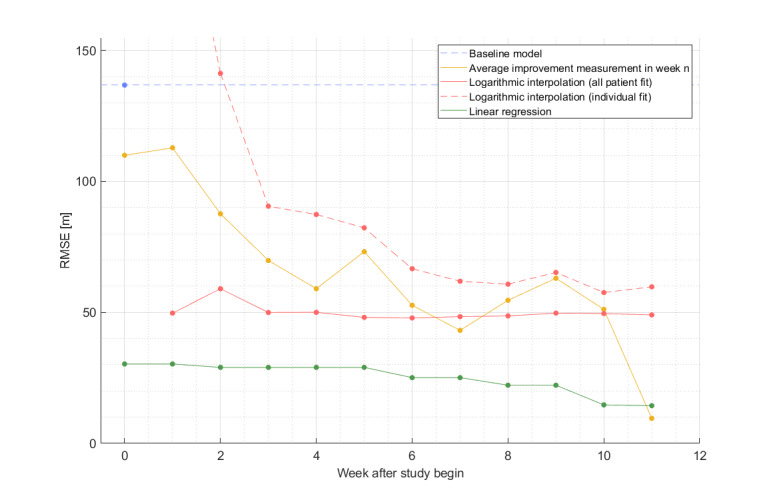
Course over time of the root mean square error (RMSE) between estimated and measured 6-minute walk test total walking distance, depending on the week of estimation, per model.

Table 4 shows the RMSEs between estimated and measured 6MWT total walking distance at the study end (week 12), depending on the week of estimation and the respective model. The distance at the baseline model, which estimated the distance at the study end equal to the distance at baseline, revealed an error of 136 meters. The average improvement in the week n method achieved an error of 109 meters at baseline and a continuous decrease of the error up to week 7 with an error of 43 meters. The logarithmic interpolation with a patient individual fit was not able to outperform the improvement in the week n method. However, the logarithmic fit on all patients was able to outperform the improvement in the week n method from week 1 to week 6. The linear regression model used data from the baseline of the study in combination with the time-dependent features, described in the Methods ection under the subsection Predictive modeling. The linear regression model continuously decreased the prediction error from week to week since the time-dependent features became more proximate to the outcome. For this analysis, the number of features was set to be between 1 and 4, whichever combination performed the best according to Table 4. The linear regression was able to outperform the logarithmic interpolation in every week. The error of 30 meters achieved by the linear regression model at baseline was 7.04% the mean total walking distance at the study end of 425 meters. The progress of the estimation errors over time is shown in Figure 4.

**Table 4.** Root mean square error (m) between the estimated 6-minute walk test (6MWT) total walking distance at the study end and measured distance, as achieved with different models based on the available data in the corresponding week.

Model	Baseline	Week										
		1	2	3	4	5	6	7	8	9	10	11
Distance at baseline	136	—^a^	—	—	—	—	—	—	—	—	—	—
6MWT data only												
Average improvement in week n	109	112	87	69	59	73	52	43	54	63	51	9
Logarithmic interpolation for individual fit	—	233	141	91	87	82	67	62	61	65	58	60
Logarithmic interpolation for all patient fit	—	50	59	50	50	48	48	48	49	50	50	49
6MWT + other data												
Linear regression	30	30	29	29	29	29	25	25	22	22	14	14

a Not applicable.

Table 5 shows the RMSE of models using data assessed in-clinic at the baseline of the study. Only the RMSE of the best-performing feature or feature combination is shown in the table. The ideal number of features for the linear regression was 4 (distance at the onset of the study, the patients’ sex, the difference between subjectively estimated distance until the onset of pain and the actual distance, and the ratio between the 2 TUG tests, both performed at the onset).

**Table 5.** Root mean square errors (m) of the estimated 6-minute walk test total distance as compared to the actually measured distance in week 12, based on the number of features at the baseline.

Model	Number of features							
	1	2	3	4	5	6	7	8
Linear regression	52.08	38.89	31.10	30.31	30.63	30.59	31.90	33.23

## Discussion

### Principal Results

In the current work, an in-depth analysis of previously not analyzed data recorded during the Keep Pace Study was performed, in which 19 patients with peripheral arterial disease performed a 12-week walking program with telemonitoring assistance. In addition to the improved pain-free walking distance as previously published in [20], we identified significant improvements in physical activity scores during the study, based on both the TUG test and the 6MWT. The regular recording of 6MWTs by the patients at home in combination with the telehealth service allowed HCPs to monitor the individual success of the SWT in real time. Based on these data (Figure 1), we identified that the majority of the 6MWT improvement happened in the first 3 weeks of the program, while saturating thereafter, which is in good accordance with the training effect in other studies, even though this stagnation of improvement happens later in healthy individuals [23]. We also identified a significant reduction of pain during the program. In contrast to the training effect, this improvement did not show any saturation but spread until the end of the program.

Despite the pain that was reported by the patients, the overall adherence to the 6MWT measurement protocol was high (85.53%). Unfortunately, we were only able to measure the adherence to the test protocol and not the adherence to the actual SWT, which was not recorded during the Keep Pace study. Since we had no insight into whether the patients continuously wore their smartphones with the step count, we could not use the daily steps to draw conclusions about the adherence to the WT. However, based on the high adherence to the measurement protocol and the overall improvement in walking distance, we assume that most patients were adherent to the training program, too. Neither adherence nor improvement during the program was dependent on the initial 6MWT distance, which indicates the program’s potential across all baseline fitness levels.

While both the 6MWT distance and the TUG time improved during the program, no significant increase in the number of steps per day, as another measurement of the daily physical activity, was observed. However, since steps were tracked by the smartphones’ native apps only, and since smartphones are not expected to be carried continuously, the number of steps might not be representative.

The TUG test is currently not a primary assessment tool for physical fitness in patients with peripheral arterial disease. However, TUG times and 6MWT distances are highly correlated in patients with peripheral arterial disease. Since the TUG test takes only approximately 20‐30 seconds (as compared to the 6 minutes of the 6MWT), it is much less burdensome and time-consuming. Further studies are required to analyze whether parts of the 6MWT can be replaced by TUG tests or if TUG data can provide additional insights in combination with 6MWTs to optimize peripheral arterial disease management.

The data showed that the physical activity and fitness levels decreased with an increase in the pain sensation, as to be expected since pain is a limiting factor for physical activity. Similarly, the general well-being correlated with the outcome of the 6MWTs. However, due to the highly unequal distribution of subjective well-being on days on which a 6MWT was performed, the correlation between well-being and 6MWT total distance must be interpreted with care. Additionally, using a scale from 1 to 3 for measuring the well-being might result in inaccurate depictions due to a lack of granularity in the reporting compared to a continuous scale from 0 to 10 which was used for the measurement of pain. This lack of granularity might explain why no significant change in the well-being during the study has been identified.

The developed predictive models highlighted that—based on telehealth data—the outcome can be predicted with an error of 30 meters at baseline, which corresponds to 7.04% of the mean total walking distance at the study end. Whether this prediction is accurate enough to help adjust the training to the individual patient or to apply patient-specific measures, such as additional phone calls, must be evaluated separately in a prospective setting. With the modeling approaches, we have shown 2 potential use cases, where (1) a prediction at study baseline can be done accurately to identify patients with great potential for the system and (2) continuous, weekly predictions to give patient-specific feedback. A prediction of the final walking distance as in case (1) could be used to not only identify patients with high potential to benefit from the system but also to motivate them to join the system by providing them with a probable result if they were adherent to the program. The predictions of (2) could be used to provide patient-specific feedback and training regime adaptions.

### Limitations

The results of this study must be interpreted with care since our dataset contained only 19 patients. Additionally, the study population was very homogeneous with only patients with peripheral arterial disease of Fontaine stage IIb since this stage benefits the most from SWT. While this work showed that predictive modeling and trend estimation work well for patients with Fontaine stage IIb, future work must validate these findings for other stages of peripheral arterial disease. Additionally, with a rather short follow-up time of 90 days, the effects of the telehealth solution on the long-term adherence to the walking program could not be evaluated.

These limitations apply especially for the predictive models. Therefore, we applied a leave-one-out train-test split to maximize the available training data and limited the number of features for the models. In general, the trend estimation worked well compared to the distance-at-baseline method but showed great differences in the RMSE between the methods.

As expected, telehealth data can improve the accuracy of models that predict 6MWT distances at the study end. Simple models showed high instability when applied on data of the first weeks only, especially when based on individual patients’ data only. However, even these models improved when telehealth data from further weeks were included. The lowest error of all models was achieved with the average improvement model applied in week 11. However, this approach performed worse in previous weeks, and predictions in week 11 might be of limited interest in a real-world application. Additionally, we assume that this extraordinarily good result might have been achieved by chance, due to the small sample size. The algorithms used for predicting the total walking distance at the study end were low in complexity due to the small study size of only 19 patients. A random forest was also tested as a possible approach but was outperformed by linear regression in every instance. More complex models like the random forest or support vector machines, or even (deep) neuronal networks, might perform even better. However, much larger datasets will be required to train these models, even if transfer learning is applied.

### Future Work

Future work should validate the effect of predictive modeling in supporting home-based structured walking training in patients with peripheral arterial disease with (a) a larger sample size, (b) prospectively, and (c) based on clinical outcomes compared to a control group only using the telehealth system without continuous predictive modeling support. Finally, our results need to be integrated into real-world telehealth settings, ideally in a prospective study to evaluate the impact of informed decision-making by the HCP based on recorded telehealth data and on the results of the predicted outcomes. This approach might not only help HCPs provide patient-specific care but could also help the patients with their motivation by seeing the progress and the prediction of what they could achieve if they stayed adherent to the walking program.

### Conclusions

This paper shows that the prototypical trend estimation has great potential for an integration in the telehealth system to be used in future work to provide tailored patient-specific advice based on these predictions. Additionally, the continuous data from the telehealth system grant a deeper insight and a better understanding of the patients’ status concerning well-being and level of pain as well as their current physical fitness level and the progress towards reaching set goals.

## Abbreviations

6MWT: 6-minute walk test
HCP: health care professional
RMSE: root mean squared error
SWT: structured walking training
TUG: timed-up-and-go
